# P-1052. Impact of Chlorhexidine Elution Profiles on Antifungal Durability of Two Chlorhexidine-Coated Antimicrobial Central Venous Catheters (CVCs)

**DOI:** 10.1093/ofid/ofaf695.1247

**Published:** 2026-01-11

**Authors:** Y Lan Truong, Joel Rosenblatt, Bahgat Z Gerges, Ying Jiang, Anne-Marie Chaftari, Ray Y Hachem, Peter Lamie, Dennis Kraus, Issam I Raad, Distinguished Professor

**Affiliations:** UT MD Anderson Cancer Center, Houston, TX; MD Anderson UT, Houston, Texas; MD Anderson UT, Houston, Texas; The University of Texas MD Anderson Cancer Center, Houston, Texas; MD Anderson UT, Houston, Texas; MD Anderson UT, Houston, Texas; UT MD Anderson Cancer Center, Houston, TX; Spectrum Vascular, Denver, Colorado; MD Anderson UT, Houston, Texas

## Abstract

**Background:**

The prevalence of Central Line Associated Bloodstream Infections (CLABSIs) due to *Candida* species continues to increase, hence CVCs with improved antifungal activities are needed. The FDA recently cleared a new antimicrobial CVC containing Chlorhexidine (C), Minocycline (M) and Rifampin (R). This makes available a second triple combination antimicrobial CVC containing antibiotics and C in addition to the widely used CVC containing C, silver (Si), and the antibiotic Sulfadiazine (Sz). Here, we measured the elution profiles over time of C from both CVCs and assessed how this impacted the antifungal efficacy and durability of the two CVCs.

**Figure:**
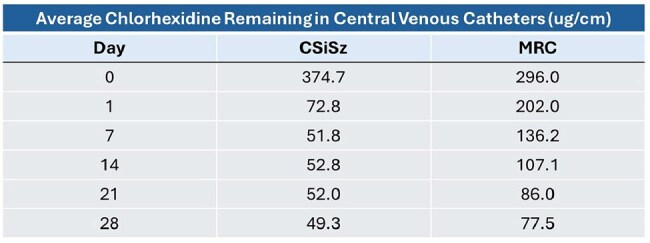
Average Amount of Chlorhexidine Remaining in the CVCs during Elution

**Figure:**
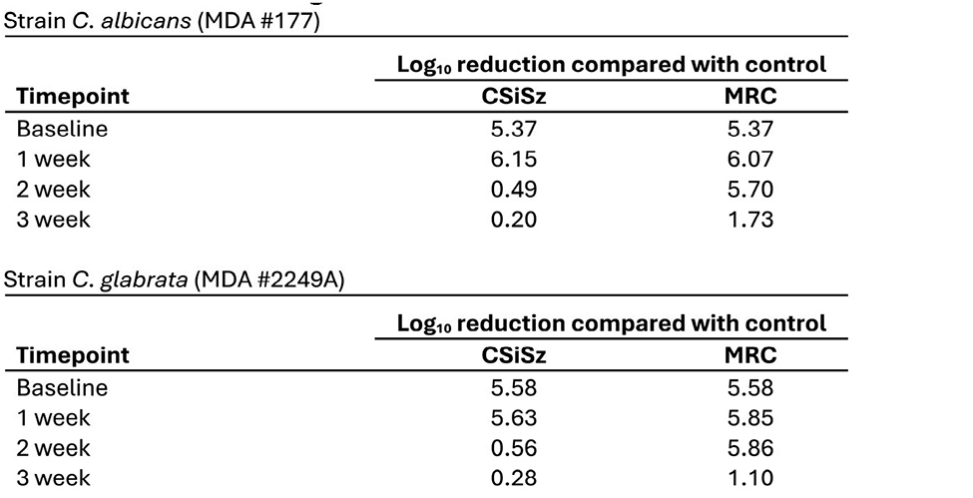
Log Reductions vs Control for Viable Colonizing C. albicans and C. glabrata Recovered from Catheters during Elution

**Methods:**

MRC CVCs were prepared by a sequential coating process. Commercially available CSiSz CVCs were purchased. CVCs were immersed in plasma for 24 hrs then immersed in serum for 3 weeks. At baseline (24 hrs) and weekly thereafter, segments were removed for chemical analysis of C content remaining by HPLC and for fungal colonization challenge. Challenge exposed the segments to inocula of clinical *Candida* CLABSI isolates for 48hrs following which the number of colonizing microbes were enumerated by sonication, serial dilution, plating and colony counting. Non-coated CVCs were controls for all testing.

**Figure:**
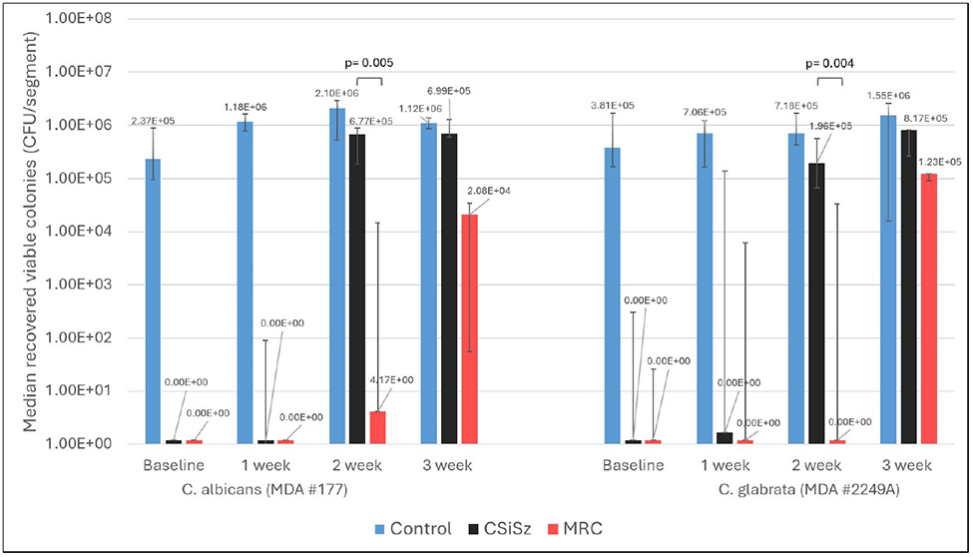
Median Number of CFU/segment Recovered following Microbial Challenges of C. albicans and C. glabrata

Statistical comparisons of CSiSz and MRC CVCs are indicated by horizontal brackets and p-values are indicated above the brackets showing whether differences were statistically significant (if p< 0.05).

**Results:**

Table 1 presents the average amount of C remaining in the CVCs as they eluted. Table 2 presents log reductions versus control for viable colonizing *C. albicans* (*CA*) and *C. glabrata* (*CG*) recovered from the catheters as they eluted. Figure 1 presents the median number of CFU/segment recovered following microbial challenges of *CA* and *CG*. The differences in antimicrobial activity between the MRC CVC and CSiSz CVC were statistically significant (p=0.005 for *CA* and p=0.004 for *CG*) at week 2 (Figure 1). The CSiSz CVC eluted approximately 80% of its starting C in the first 24 hrs and over 85% after 1 week. Very little C eluted after 1 week. The MRC CVC eluted approximately 30% of its initial C at 1 week, 50% at 2 weeks and 55% at 3 weeks.

**Conclusion:**

Corresponding to the elution differences, the MRC CVC had greater antimicrobial durability against both *Candida* strains, most pronounced at week 2.

**Disclosures:**

Joel Rosenblatt, PhD, Citius Pharmaceuticals, Inc.: Advisor/Consultant|Citius Pharmaceuticals, Inc.: Grant/Research Support|Citius Pharmaceuticals, Inc.: Patent|Citius Pharmaceuticals, Inc.: Ownership Interest|Citius Pharmaceuticals, Inc.: Stocks/Bonds (Public Company)|Spectrum Vascular: SV Spectrum MRC Central Venous Catheter; SV Spectrum MR Central Venous Catheter; SV Central Venous Catheter|Spectrum Vascular: Ownership Interest Anne-Marie Chaftari, MD, Citius Pharmaceuticals, Inc., Cranford, New Jersey, USA: Grant/Research Support Dennis Kraus, MD, Spectrum Vascular: Patent|Spectrum Vascular: Ownership Interest Issam I. Raad, Distinguished Professor, Citius Pharmaceuticals, Inc. (Grant/Research Support): Advisor/Consultant|Citius Pharmaceuticals, Inc. (Grant/Research Support): Grant/Research Support|Citius Pharmaceuticals, Inc. (Grant/Research Support): Patent|Citius Pharmaceuticals, Inc. (Grant/Research Support): Ownership Interest|Citius Pharmaceuticals, Inc. (Grant/Research Support): Stocks/Bonds (Public Company)|Spectrum Vascular: Patent|Spectrum Vascular: Ownership Interest

